# Infection by and protective immune responses against *Plasmodium berghei *ANKA are not affected in macrophage scavenger receptors A deficient mice

**DOI:** 10.1186/1471-2180-6-73

**Published:** 2006-08-16

**Authors:** Margarida Cunha-Rodrigues, Sílvia Portugal, Maria Febbraio, Maria M Mota

**Affiliations:** 1 Instituto de Medicina Molecular, Universidade de Lisboa, 1649-028 Lisboa, Portugal; 2 Instituto Gulbenkian de Ciência, Oeiras, Portugal; 3 Cellular Biology Lerner Research Institute, Cleveland, Ohio, USA

## Abstract

**Background:**

Scavenger receptors (SRs) recognize endogenous molecules modified by pathological processes as well as components of diverse microorganisms. Mice deficient for both SR-AI and II are more susceptible to infections by a variety of bacterial and viral pathogens.

**Results:**

Here we show that SR-A deficient mice and wild type mice are equally susceptible to malaria infection both during liver and blood stages. Moreover, like wild type mice, SR-A deficient mice are able to mount a protective immune response against radiation attenuated sporozoites.

**Conclusion:**

Our results do not reveal a function of SR-A I and II receptors in the *Plasmodium berghei *ANKA infection, both in the development of CM and parasitemia control. Moreover, these receptors appear not to be required for the establishment of a protective immune response against the malaria liver stages.

## Background

Malaria infection starts in the mammalian host with the injection of *Plasmodium *sporozoites by a mosquito bite. Sporozoites travel to the liver where they cross the sinusoidal wall through Kupffer cells and then migrate through several hepatocytes before they establish an infection with the formation of a parasitophorous vacuole [1,2]. Within the vacuole the sporozoites develop and generate millions of merozoites that are released into the bloodstream. With the infection of erythrocytes the clinical phase of malaria begins. In any given year, more than a million children die as a result of malaria infection. The death from infection is largely due to an acute syndrome known as cerebral malaria (CM). The neurological manifestations of CM include headache, agitation, psychosis, seizures and impaired consciousness that lead to coma and death [3].

The class A macrophage scavenger receptor (SR-A) is the prototypic member of a large family of membrane receptors that bind oxidized low density lipoprotein and a wide variety of other ligands many of which are derived from apoptotic cells and pathogens [4]. The SR-A receptors occur in two different forms that are generated by alternative splicing of the primary transcript: SR-AI and SR-AII. Both receptors have nearly identical ligand binding properties [4]. They are expressed primarily by mature cells of the myelomonocytic lineage such as Kupffer cells in the liver, glial cells in the brain and macrophages that are resident in or recruited to various tissues. They are also expressed by sinusoidal endothelial cells in the liver [5]. SR-A receptors appear to have beneficial and pathological functions. They mediate phagocytosis of apoptotic cells [6], and have been implicated in atherogenesis, the clearance of debris during acute neuronal degeneration [7] as well as in innate immunity and antigen presentation [6]. SR-A receptors bind lipopolysaccharide from Gram-negative and lipoteichoic acid from Gram-positive bacteria [4]. SR-AI and II receptor deficient mice (SR-A^-/-^) are more susceptible to infections by a variety of pathogenic microorganisms such as *Listeria monocytogenes *[8], *Staphylococcus aureus *[9], *Bacillus Calmette-Guérin *[10] and herpes simplex virus [8].

In the present work we examined the role of SR-A receptors in three different aspects of a malaria infection, namely first, in the establishment of a primary liver infection; second, in the protective immune response against the liver stages; and third, in the development of the cerebral pathology associated with blood stages of infection.

## Results and discussion

**Figure 1:**
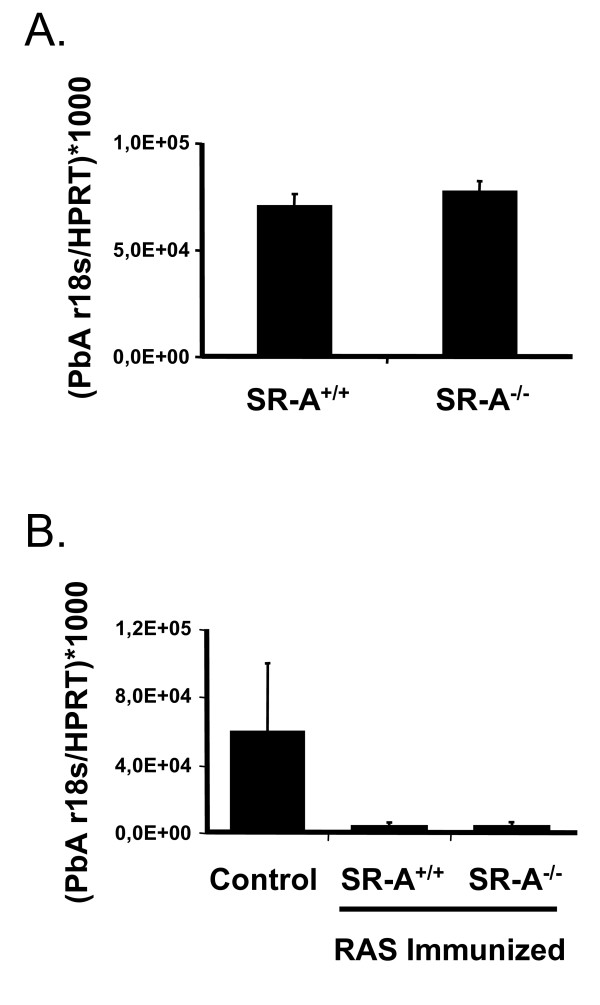
**Lack of SR-A expression does not affect *Plasmodium berghei *liver infection. **(A) *Plasmodium berghei *quantification in the liver 40 hours post infection with *Plasmodium berghei *ANKA sporozoites as measured by qRT-PCR. (B) *Plasmodium berghei *quantification in the liver 40 hours post challenge with 10,000 *Plasmodium berghei *ANKA sporozoites, in animals previously immunized with a single dose of 50,000 radiation attenuated *Plasmodium berghei *ANKA sporozoites (RAS), as measured by qRT-PCR. Shown is the mean number of *Plasmodium berghei *ANKA 18s per HPRT mRNA molecules (×10^3^) ± standard deviation (n = 4 mice per plot). Experiments were repeated twice.

The initial targeting of sporozoites to the liver involves close interactions with sinusoidal endothelial cells and Kupffer cells [2], both of which express SR-AI and SR-AII receptors. To study the possible involvement of these receptors in the initial stages of malaria infection we infected SR-A^-/- ^mice as well as their SR-A^+/+ ^littermates [8] with 2 × 10^4 ^*Plasmodium berghei *ANKA sporozoites. The parasite burden in the liver was measured 40 hours after infection, just prior to the release of merozoites into the bloodstream. The level of infection was determined by quantitative RT-PCR (qRT-PCR) using specific primers for *Plasmodium berghei *18s ribosomal RNA [11]. There was no difference in the level *Plasmodium berghei *ANKA infection in SR-A^-/- ^mice, when compared to SR-A^+/+ ^mice (Fig. 1A).

SR-A receptor functions have been implicated in both the innate and the adaptive branches of the immune response. To determine whether SR-A receptors play a role in immune responses against malaria, we used a well-established model of immunization against the liver stages of malaria infection. Inoculation with radiation attenuated sporozoites (RAS) results in protection against a subsequent challenge with infective sporozoites, both in mice and humans [12,13]. Irradiated sporozoites invade hepatocytes but do not develop normally and thus fail to establish an erythrocytic infection [14]. SR-A^-/- ^mice as well as their SR-A^+/+ ^littermates [8] were immunized with 5 × 10^4 ^*Plasmodium berghei *RAS and challenged 10 days later with 10^4 ^infective sporozoites. Forty two hours after the challenge the level of protection was measured by quantifying the parasite load in the liver using qRT-PCR, as described above. RAS immunization of SR-A^+/+ ^mice, as expected, clearly reduced the level of infection measured 40 hours after a challenge with infective sporozoites. The same level of protection was achieved in SR-A^-/- ^mice (Fig. 1B). This shows that the lack of SR-A receptors does not affect the establishment of a protective immune response against RAS.

**Figure 2:**
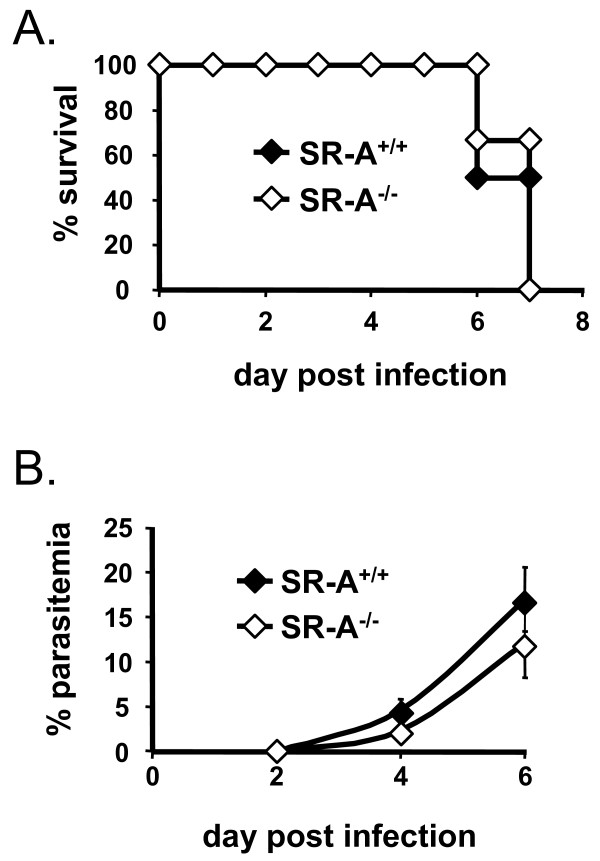
**Lack of SR-A expression does not affect *Plasmodium berghei *infection and its associated pathology. **Survival (A) and parasitemia (B) of SR-A^-/- ^and SR-A^+/+ ^mice infected with 10^6 ^*Plasmodium berghei *ANKA infected red blood cells. Mean ± standard deviation (n = 4 mice per plot) is shown for parasitemias. Experiment repeated 3 times.

The molecular and cellular mechanisms that lead to the devastating symptoms of CM remain poorly understood. The disease is associated with enhanced adherence of infected erythrocytes to the cerebral vasculature, disruption of the blood-brain barrier, activation and proliferation of migroglial cells, expression and secretion of proinflammatory molecules, and recruitment of macrophages and other inflammatory cells to the primary lesions. SR-AI and II receptors are expressed by both activated macrophages and glial cells. They could have disease promoting or protective functions. Infection of C57BL/6 mice with *Plasmodium berghei *ANKA is a well-established experimental model of malaria infection. When infected with *Plasmodium berghei *ANKA, C57BL/6 mice develop neurological disturbances such as hemi- or paraplegia, deviation of the head, tendency to roll over on stimulation, ataxia and convulsions and die between days 6 to 9 post-infection [15]. Due to the many similarities with human CM this neurological syndrome is referred to as experimental CM (ECM) (*reviewed in *[15]). Still, caution should be taken when extrapolating from the rodent model to the human disease. While human CM is predominantly associated with iRBC adherence to the microvasculature of the brain, mouse brains infected with *P. berghei *ANKA (and showing symptoms of ECM) show blood brain vessel congestion containing mainly leucocytes and low level of iRBCs. Despite these differences, the altered cells that play a pivotal role in human and rodent lesions during malaria infection are the same, the brain microvascular endothelial cells. We inoculated both SR-A^-/- ^mice and their SR-A^+/+ ^littermates in C57BL/6 background with 10^6 ^*Plasmodium berghei *ANKA infected erythrocytes. All mice in both groups developed neurological symptoms and died between 6 to 8 days after infection (Fig. 2A). There were no significant differences in the level of parasitemia between the 2 groups (Fig. 2B). The results were similar when infection was initiated with 2 × 10^4 ^sporozoites and allowed to proceed for blood stages (data not shown). This finding is in agreement with a short report by Nogami *et al*. who found the same levels of parasitemia in SR-A deficient and wild type mice after inoculation with erythrocytes that were infected with *Plasmodium berghei *NK65, an experimental model that does not induce CM [16]. In that study only 1 out of 8 SR-A^+/+ ^mice died on day 10, while in the group 2 out of 8 mice died on day 7 and a further 3 mice died on day 17. Since these deaths coincided with the two peaks of parasitemia, the authors suggested that SR-A^-/- ^mice might have more difficulty in controlling peaks of parasitemia. Thus, we sought to determine the role of SR-A in the course of infection of a non-lethal malaria rodent model, *P. yoelii *17×. In this model, wild-type mice produce a peak of parasitemia around day 10 post-infection and are able to control it with 100% survival and full parasite clearance. SR-A^-/- ^infected in parallel showed no difference in survival or parasitemia during the course of infection (data not shown), again in agreement with the results very recently published by Inoue *et al*. [17].

Taken together our data suggests that the lack of SR-A receptors has no influence in the malaria outcome during the blood stages of infection.

## Conclusion

The results shown here do not reveal a function of SR-A I and II receptors in the *Plasmodium berghei *ANKA infection, in the development of CM or parasitemia control. Moreover, these receptors appear not to be required for the establishment of a protective immune response against the malaria liver stages in the well-established model of immunity against RAS. It has been shown that mice deficient in another member of the scavenger receptor family, CD36, have no differences at the liver infection [18]. Moreover, recent experiments with mice deficient in CD36, indicate that this receptor is involved in the sequestration of *Plasmodium berghei *ANKA to the lung and adipose tissues but not in the development of cerebral malaria [19]. In view of the complex and overlapping functions of different scavenger receptors it is conceivable that the true role of individual receptors is not revealed in experiments with receptor deficient mice unless a specific function of the receptor is analyzed [6]. Whether SR-A receptors do have such specific functions in malaria infections of mice or humans remains to be elucidated.

## Methods

### Infection

*Plasmodium berghei *ANKA sporozoites were obtained from dissection of infected *Anopheles stephensi *mosquito salivary glands from the IMM insectary production. Male, 7–9 weeks old, SR-A^-/- ^and their control littermate mice generously provided by Dr. T. Kodama (University of Tokyo) [8] were infected by intravenous injection of *Plasmodium berghei *(2 × 10^4^) sporozoites or by intraperitoneal injection of *Plasmodium berghei *ANKA or *P. yoelii *17 × (10^6^) infected erythrocytes (originally, provided by Dr. A. Waters and C. Janse, Leiden University, or Dr. David Walliker, Edimburgh University, respectively).

### Parasite quantification in the liver

40 hours post infection with sporozoites livers were removed, homogenized and total RNA was extracted (RNeasy Mini kit Quiagen). cDNA was obtained by reverse transcription (First-strand cDNA synthesis kit, Roche). Real-time PCR, using primers specific for *Plasmodium berghei *18S rRNA (5'-AAGCATTAAATAAAGCGAATACATCCTTAC-3' and 5'-GGAGATTGGTTTTGACGTTTATGTG-3'), was used for quantification of parasite load in the livers of mice 40 hours after sporozoite inoculation.

### Immunization protocol

Immunizations with radition attenuated sorozoites was perfomed by injecting 5 × 10^4 ^γ-irradiated (16,000 rads) *Plasmodium berghei *ANKA sporozoites followed by challenge (10 days later) with 10^4 ^infective sporozoites, both by intravenous injection. Parasite burden in the liver was measured as described in the previous section.

### Following blood stage infection and pathology

Cerebral complications were monitored by ataxia, paralysis, deviation of the head and convulsions, symptoms leading to coma and death. Blood peripheral parasitemia was determined by counting parasites in Giemsa stained thin blood films.
